# Sensitivity of Murphy's sign on the diagnosis of acute cholecystitis: is it really so insensitive?

**DOI:** 10.1002/jhbp.657

**Published:** 2019-07-25

**Authors:** Ryohei Sekimoto, Kentaro Iwata

**Affiliations:** ^1^ Kobe University School of Medicine Kobe Hyogo Japan; ^2^ Kobe University Graduate School of Medicine Kobe Hyogo Japan

Dear Editor,

Tokyo Guideline 2018 states Murphy's sign is not sensitive, citing a single center study with sensitivity of only 20.5% 1, 2. However, a systematic review and an evidence‐based review of physical diagnosis stated the contrary, with sensitivity ranging 58–71% and 48–97%, respectively 3, 4. We believe that clinical guidelines should cite references in a systematic, comprehensive, and impartial manner. Citing a single article using data from a single center only, while ignoring other studies with better sensitivity could misguide the readers of the guideline. We would like to request the authors of the guideline the comprehensive literature review and fair citation of the clinical data, with reasonable revision of the manuscript upon the next latest guideline.

## Conflict of interest

None declared.
